# Incomplete Penetrance of Population-Based Genetic Screening Results in Electronic Health Record

**DOI:** 10.3389/fgene.2022.866169

**Published:** 2022-04-27

**Authors:** Gai Elhanan, Daniel Kiser, Iva Neveux, Shaun Dabe, Alexandre Bolze, William J. Metcalf, James T. Lu, Joseph J. Grzymski

**Affiliations:** ^1^ Center for Genomic Medicine, Desert Research Institute, Reno, NV, United States; ^2^ Renown Health, Reno, NV, United States; ^3^ Helix, San Mateo, CA, United States

**Keywords:** CDC Tier 1, HBOC, BRCA, EHR, Lynch, familial hypercholesterolemia, genetic screening, population health

## Abstract

The clinical value of population-based genetic screening projects depends on the actions taken on the findings. The Healthy Nevada Project (HNP) is an all-comer genetic screening and research project based in northern Nevada. HNP participants with CDC Tier 1 findings of hereditary breast and ovarian cancer syndrome (HBOC), Lynch syndrome (LS), or familial hypercholesterolemia (FH) are notified and provided with genetic counseling. However, the HNP subsequently takes a “hands-off” approach: it is the responsibility of notified participants to share their findings with their healthcare providers, and providers are expected to implement the recommended action plans. Thus, the HNP presents an opportunity to evaluate the efficiency of participant and provider responses to notification of important genetic findings, using electronic health records (EHRs) at Renown Health (a large regional hospital in northern Nevada). Out of 520 HNP participants with findings, we identified 250 participants who were notified of their findings and who had an EHR. 107 of these participants responded to a survey, with 76 (71%) indicating that they had shared their findings with their healthcare providers. However, a sufficiently specific genetic diagnosis appeared in the EHRs and problem lists of only 22 and 10%, respectively, of participants without prior knowledge. Furthermore, review of participant EHRs provided evidence of possible relevant changes in clinical care for only a handful of participants. Up to 19% of participants would have benefited from earlier screening due to prior presentation of their condition. These results suggest that continuous support for both participants and their providers is necessary to maximize the benefit of population-based genetic screening. We recommend that genetic screening projects require participants’ consent to directly document their genetic findings in their EHRs. Additionally, we recommend that they provide healthcare providers with ongoing training regarding documentation of findings and with clinical decision support regarding subsequent care.

## Introduction

Population-based genetic screening (PbGS) can be a valuable risk assessment tool for relatively common genetic conditions with high penetrance such as hereditary breast and ovarian cancer (HBOC), Lynch syndrome (LS) and familial hypercholesterolemia (FH) (Tafe, 2015; Lambert et al., 2019; Evans et al., 2020; Manchanda et al., 2020; Patel et al., 2020; Ficarazzi et al., 2021). Many individuals at-risk for these conditions are not identified by current medical practices (Manickam et al., 2018; Grzymski et al., 2020; Murray et al., 2020; Patel et al., 2020) and their family members may benefit from cascade genetic screening (George et al., 2015; Patel et al., 2020). However, screening the general population can only be effective if genetic findings are successfully disseminated to project participants and if a significant portion of the screened individuals follow recommended actions. However, this may not necessarily be the case as it has been shown that the uptake of genetic testing and their results may be sub-optimal and that primary care providers are still not comfortable with genetic testing (Press et al., 2000; Binetti et al., 2006; Finlay et al., 2008; George et al., 2015; Godino et al., 2016; Bijlsma et al., 2018; Menko et al., 2019; Actkins et al., 2021; David et al., 2021).

Not all PbGS projects are alike, and their underlying design may affect the dissemination and uptake of the genetic findings. The Healthy Nevada Project (HNP) (Grzymski et al., 2020; Read et al., 2021) is an all-comer health determinants PbGS research project based in northern Nevada. The second phase of the HNP provides clinical exome sequencing (Helix, 2017) for all participants, of which there are currently 45,000 (roughly 5 percent of the regional population). HNP participants are asked for three levels of consent: consent to 1) provide a saliva sample, 2) receive notification of positive findings and genetic consultation and 3) participate in further research. Only the first consent is required to participate in the HNP. As previously described (Grzymski et al., 2020), more than 99 percent of participants consented to receive notification of positive findings and consultations by licensed genetic counselors (LGCs) for three CDC Tier 1 conditions (Centers for Disease Control and Prevention, 2021; Miller et al., 2021) (T1pos) with a potential for individual and population health benefit: HBOC, LS and FH. LGCs attempt to contact each T1pos participant up to six times based on the preferred contact method(s) provided at the time of consent. Once T1pos participants have been successfully contacted, the LGCs explain the significance of each participant’s finding and outline what the participant should do next. Next steps include obtaining confirmatory testing, notifying the participant’s primary care physician (PCP) of the findings, and formulating an appropriate action plan with their PCP. Other than direct contact with the LGCs, no alternative notification methods were employed, and for the results presented in this study, the HNP did not directly update the participant’s electronic health record (EHR) with their genetic findings and results were not directly accessible to physicians or other healthcare personnel. The HNP does not notify participants regarding absence of findings. While sequencing was performed by a CLIA-certified lab, interpretations were performed by HNP personnel (Grzymski et al., 2020) during the initial phase of the HNP. Therefore, confirmatory testing was required as part of the project protocol. Later, interpretations were provided by a CLIA-certified lab, but the requirement for confirmatory testing remained as part of the protocol.

The HNP is supported by Renown Health^1^ (Renown), the largest healthcare provider in northern Nevada. Since Renown provides nearly 70 percent of the inpatient care and about 50 percent of primary care in the region, its EHR offers an opportunity to examine the effect of returning actionable genetic findings on the diagnoses recorded and the clinical actions subsequently taken by the participants and their healthcare providers.

We report here the effect of returning genetic findings on diagnoses and clinical actions recorded in the Renown EHR for HBOC, LS and FH T1pos participants in the HNP.

## Methods

For details of the HNP and definitions of pathogenic and likely pathogenic T1pos findings please see (Grzymski et al., 2020).

We conducted a comprehensive electronic review of extracted data from T1pos participants’ Renown’s EHRs (including clinical notes). We also reviewed responses from a survey sent to all T1pos consenting participants regarding delivery of findings and follow-up actions.

### EHR Review

EHR data were available from Renown via the Epic^2^ Clarity database, a large subset of the data in the Epic EHR application. EHR data were available from 2006 to 23 August 2021, although the EHR wasn’t fully implemented until 2011. The patient diagnosis data review was conducted in June 2021 and participants were included for EHR review if at least 3 months passed since the T1pos notification to ensure that participants had time to respond to their findings.

Diagnoses were retrieved using native application diagnosis codes (nDx) found in more than forty clinical and administrative/billing tables. Each nDx was associated with an entry date as well as a native diagnosis description and mapping (if available) to ICD-9-CM^3^ and/or ICD-10-CM^4^ codes. In general, nDxs map to one or more ICD codes and are often more specific than ICD codes. Because of their greater specificity, we used nDxs for our analysis rather than ICD codes.

All retrieved nDxs were initially reviewed based on their description and only diagnoses deemed relevant to the T1pos finding of an individual were retained (Supplementary Material S1). A detailed review of the remaining nDxs was conducted to determine relevance to each specific T1pos condition. All nDxs reviews were conducted by a physician (GE) with an Internal Medicine background. Prior knowledge of T1pos conditions was defined as a genetic diagnosis appearing in the EHR prior to the notification date.

For ancillary procedures, we focused on retrieving representative screenings for each condition: mammograms and other types of screening breast imaging procedures for HBOC (Winters et al., 2017; Lee et al., 2020), colonoscopies for LS (Jasperson et al., 2010; Peterse et al., 2020) and LDL tests for FH (Youngblom et al., 2014; Jellinger et al., 2017). LDL laboratory results were retrieved directly from the results table in Clarity based on native component codes, whereas imaging and other procedures were collected based on orders, results, and mentions in the clinical notes. We also retrieved indications that a mastectomy or oophorectomy was performed from diagnosis tables, clinical notes, and surgery log tables.

Clinical notes for all participants with medical records were retrieved based on a comprehensive keyword search using terms related to each individual condition (HBOC, LS, FH); to genetic testing, findings, or consultations; and to the HNP. Several iterations of the keyword search term collection were performed until no missed terms were found in two repetitive random samples of 100 notes from the entire collection of T1pos participants’ notes. All selected notes were then manually reviewed by a single reviewer (GE) for any references pertinent to T1pos findings.

To determine whether participants and their physicians possibly enacted changes to clinical care after notification, we visually examined patient timelines. Changes in care were suspected under the following conditions: if there was an increase in the frequency of mammographies or if prophylactic mastectomies or oophorectomies were performed (HBOC), if a new colonoscopy was ordered without prior history of screening colonoscopy or outside of the recommended timeframe of repeat colonoscopy (LS), for FH we used change in LDL levels as an overall indicator of lifestyle changes and outcome of possible prescribing of effective lipid lowering medications.

Explicit referrals for confirmatory genetic testing were not visible from the Renown EHR. However, we examined recorded referrals for LGCs within the Renown EHR as well as available data from the third-party vendor^5^ that conducted genetic consultations on behalf of the HNP and was responsible for such recommendations for confirmatory testing.

### Survey

Surveys were sent in January 2020 and October 2021 to 462 T1pos participants that had consented to further research participation (not all were included in our study due to a cutoff point of May 2021 for T1pos results). The survey (Supplementary Material S2) was electronic, and participants answered up to 24 questions, depending on their responses. Several reminders were sent within 2 weeks to participants who had not yet responded to the survey. Survey responses were then aggregated and analyzed, and the responses of participants who were also Renown patients were matched with their EHR.

### Statistical Analysis

Most results reported in this study were descriptive and did not require the use of statistical tests. However, Fisher’s Exact Tests were used to test whether the likelihood that a participant was T1pos, was notified, or had an EHR record differed due to sex or race, and Wilcoxon Rank Sum Tests were used to test whether there were differences due to age. Pearson’s Chi-squared Tests were used to test whether survey responses differed between T1pos conditions. A Bonferroni correction was used to adjust for multiple testing where appropriate.

## Results

### Description of Study Participants

**FIGURE 1:**
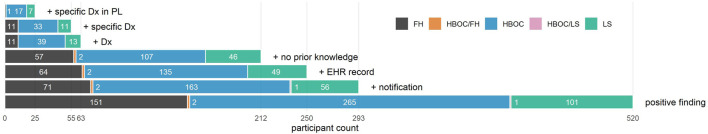
Bar graph depicting counts of participants who meet increasingly restrictive criteria. From bottom to top, participants are limited to (1) those who had a positive finding for HBOC, LS, and/or FH; (2) those who were also notified of their finding and had a genetic consultation; (3) those who also had an EHR record at Renown; (4) those who also had no knowledge of their finding documented in their EHR prior to notification; (5) those who also had a relevant genetic diagnosis documented in their EHR after notification; (6) those whose diagnosis was specific to their condition; and (7) those whose diagnosis appeared in their problem list. Total participant counts for each additional criterion appear on the *x*-axis, while counts for each distinct condition (or set of conditions) are superimposed on each bar.

On May 2021 there were 520 HNP participants (out of 41,835) that were T1pos for HBOC (268 participants), LS (102 participants) and/or FH (153 participants) (Figure 1). There were two participants with both HBOC and FH and one participant with both HBOC and LS. Participants in this study were notified between September 2018 and September 2020. Notification and counseling were completed for 293 (56.3%) of the 520 T1pos participants, and notification success was significantly higher for white participants (Table 1).

**TABLE 1:** Demographic statistics associated with Tier 1 status, notification status, and whether a participant had an EHR record. Comparisons of age, sex, and ethnicity were made for all Tier 1 conditions combined, and for HBOC, LS, and FH participants separately.

	N (%)	Age, Mean (SD)*	Female, n (%)**	White, n (%)**	Missing Demographic Data, n (%)
HNP	41835 (100.0%)	51.7 (17.2)	27836 (66.6%)	33958 (81.3%)	47 (0.1%)
All Tier 1 conditions					
HNP					
Tier 1 positive	520 (1.2%)	50.1 (17.2)	343 (66.1%)	429 (82.7%)	1 (0.2%)
Tier 1 negative	41315 (98.8%)	51.7 (17.2)	27493 (66.6%)	33529 (81.2%)	46 (0.1%)
*p*-values		0.0458	0.8149	0.4287	
Tier 1 positive					
notified	293 (56.3%)	50.1 (17.7)	194 (66.2%)	261 (89.1%)	0 (0.0%)
not notified	227 (43.7%)	50.1 (16.6)	149 (65.9%)	168 (74.3%)	1 (0.4%)
*p*-values		0.9025	1.0000	**0.0000** ^†^	
Tier 1 positive + notified					
EHR	250 (85.3%)	50.5 (17.9)	166 (66.4%)	228 (91.2%)	0 (0.0%)
no EHR	43 (14.7%)	47.5 (17.0)	28 (65.1%)	33 (76.7%)	0 (0.0%)
*p*-values		0.3294	0.863	0.0136	
Hereditary Breast and Ovarian Cancer Syndrome					
HNP					
HBOC positive	268 (0.6%)	49.1 (17.1)	166 (62.2%)	225 (84.3%)	1 (0.4%)
HBOC negative	41567 (99.4%)	51.7 (17.2)	27670 (66.6%)	33733 (81.2%)	46 (0.1%)
*p*-values		0.0183	0.1342	0.2376	
HBOC positive					
notified	166 (61.7%)	49.0 (17.2)	102 (61.4%)	144 (86.7%)	0 (0.0%)
not notified	102 (37.9%)	49.3 (17.0)	64 (63.4%)	81 (80.2%)	1 (1.0%)
*p*-values		0.7518	0.7955	0.1681	
HBOC positive + notified					
EHR	137 (82.5%)	50.0 (17.1)	85 (62.0%)	123 (89.8%)	0 (0.0%)
no EHR	29 (17.5%)	44.1 (17.3)	17 (58.6%)	21 (72.4%)	0 (0.0%)
*p*-values		0.093	0.8341	0.0293	
Lynch Syndrome					
HNP					
LS positive	102 (0.2%)	52.1 (17.9)	72 (70.6%)	85 (83.3%)	0 (0.0%)
LS negative	41733 (99.8%)	51.7 (17.2)	27764 (66.6%)	33873 (81.3%)	47 (0.1%)
*p*-values		0.8365	0.4619	0.7031	
LS positive					
notified	57 (55.3%)	51.6 (18.7)	41 (71.9%)	52 (91.2%)	0 (0.0%)
not notified	45 (43.7%)	52.7 (17.0)	31 (68.9%)	33 (73.3%)	0 (0.0%)
*p*-values		0.8031	0.8278	0.0301	
LS positive + notified					
EHR	49 (86.0%)	51.2 (19.1)	36 (73.5%)	45 (91.8%)	0 (0.0%)
no EHR	8 (14.0%)	54.1 (16.6)	5 (62.5%)	7 (87.5%)	0 (0.0%)
*p*-values		0.8094	0.6735	0.5446	
Familial Hypercholesterolemia					
HNP					
FH positive	153 (0.4%)	50.4 (16.9)	106 (69.3%)	121 (79.1%)	0 (0.0%)
FH negative	41682 (99.6%)	51.7 (17.2)	27730 (66.6%)	33837 (81.3%)	47 (0.1%)
*p*-values		0.3932	0.5478	0.4686	
FH positive					
notified	73 (47.4%)	51.2 (18.1)	52 (71.2%)	67 (91.8%)	0 (0.0%)
not notified	80 (51.9%)	49.7 (15.8)	54 (67.5%)	54 (67.5%)	0 (0.0%)
*p*-values		0.6323	0.726	**0.0003** ^†^	
FH positive + notified					
EHR	66 (90.4%)	50.7 (18.6)	46 (69.7%)	61 (92.4%)	0 (0.0%)
no EHR	7 (9.6%)	55.3 (11.5)	6 (85.7%)	6 (85.7%)	0 (0.0%)
*p*-values		0.4593	0.665	0.4663	

* Test of statistical difference was Wilcoxon Rank Sum Test.

** Test of statistical difference was Fisher’s Exact Test.

† Statistically significant after Bonferroni correction, *p* < 0.0014.

Out of the 520 T1pos participants, 417 had reviewable EHRs. After filtering out diagnoses clearly unrelated to HBOC, LS or FH, 14,584 nDXs were collected for those 417 participants (corresponding to an average of 35 unique native diagnoses per participant). 250 (60%) of the 417 participants with Renown EHR were successfully notified. Their mean age was 47.5, they were 33.2% male, and they had a total of 9,034 nDXs, or an average of 36 unique nDXs per individual. All notified participants with EHR record met the minimum required 3 months time span between notification and EHR review (mean 2.2 years, minimum 0.9 years, maximum 2.9 years). 41 of these participants had EHR records with 20 or less nDXs, while mean time span for nDXs was 8.6 years (standard deviation: 6.0 years). Therefore, none of these participants were excluded due to lack of follow-up.

Among T1pos participants with reviewable EHRs, there were 72 out of 417 individuals with malignancies typically associated with HBOC or LS. Fifty such malignancies occurred prior to the initiation of the HNP in 2018 and only five individuals were referred to genetic consultation. Three of the five had meaningful related family history documented in the EHR prior or around the time of the diagnosis of malignancy. Sixteen participants were diagnosed with HBOC/LS typical malignancies after 2017 and prior to notification by the HNP of their T1pos findings. Five of them were referred to a LGC, three of them with strong family history documented at the time of or prior to the cancer diagnosis.

### Genetic Diagnoses in the EHR

Based on review of genetic diagnoses among the 250 notified participants with EHR, 38 (15%) had EHR evidence that knowledge of their condition preceded notification, while 212 had no evidence of prior knowledge in their EHR (Figure 1). 47 (19%) could have benefited from earlier notification due to prior presentation of disease (27 HBOC, 9 LS, and 11 CVD before the age of 50 years).

Many of the genetic nDxs were non-specific even though specific nDxs, including some mentioning specific variants, exist in the system (Table 2). The four most frequent nDxs were non-specific and map directly to correspondingly non-specific ICD-10-CM codes. For HBOC, the nDx often indicated only breast or ovarian cancer susceptibility rather than susceptibility to all cancers associated with HBOC. Of the 212 participants who were notified, had an EHR, and did not have prior knowledge, 63 (30%) had at least a non-specific genetic diagnosis in their EHR, 55 (26%) had a specific genetic diagnosis in their EHR, and 25 (12%) had a specific genetic diagnosis listed in their problem list (PL) (Figure 1). We also noted that among more than 35,000 HNP participants with EHR records, 354 have a diagnosis of “Familial Hypercholesterolemia”. However, 316 of these participants were not T1pos. Also, only 11 (19%) of the 59 FH-notified participants without prior knowledge had a specific FH diagnosis, and only one of these diagnoses appeared in the PL. Review of the clinical notes of the 354 HNP participants with an FH diagnosis found only a single case where the clinical FH diagnosis was supported by a documented Dutch Lipid Clinic Network Criteria (DLCN). No evidence of use of the Simon Broome or the Making Early Diagnosis Prevents Early Death (MEDPED) clinical criteria was found.

**TABLE 2:** Relative abundance of unique diagnoses appearing in participant EHRs. Shaded diagnoses are considered to be sufficiently specific for clinical purposes.

Diagnoses	N	%	ICD-9-CM	ICD-10-CM
Genetic susceptibility to malignant neoplasm of breast	59	15.3		Z15.01
Genetic susceptibility to other malignant neoplasm	54	14.0		Z15.09
Genetic susceptibility to malignant neoplasm of ovary	39	10.1		Z15.02
Genetic susceptibility to malignant neoplasm of breast	29	7.5	V84.01	Z15.01
BRCA2 gene mutation positive in female	16	4.2	V84.01, V84.02, V84.09	Z15.01, Z15.02, Z15.09
Familial hypercholesterolemia	14	3.6		E78.01
BRCA2 positive	13	3.4	V84.01	Z15.01, Z15.09
Familial hypercholesterolemia	11	2.9	272	E78.01
Lynch syndrome	11	2.9	V84.09	Z15.09
Genetic susceptibility to malignant neoplasm of ovary	10	2.6	V84.02	Z15.02
BRCA1 positive	10	2.6	V84.01	Z15.01, Z15.09
Breast cancer genetic susceptibility	9	2.3	V84.01	Z15.01
BRCA gene mutation positive in female	9	2.3	V84.01, V84.02, V84.09	Z15.01, Z15.02, Z15.09
BRCA gene mutation positive	9	2.3	V84.01, V84.02	Z15.01, Z15.09
BRCA positive	8	2.1	V84.01, V84.02	Z15.01, Z15.09
BRCA2 genetic carrier	8	2.1	V84.01	Z15.01, Z15.09
BRCA2 gene mutation positive	8	2.1	V84.01	Z15.01, Z15.09
BRCA gene positive	7	1.8	V84.01, V84.02	Z15.01, Z15.09
Genetic susceptibility to other malignant neoplasm	6	1.6	V84.09	Z15.09
BRCA1 gene mutation positive	6	1.6	V84.01	Z15.01, Z15.09
Genetic susceptibility to malignant neoplasm of prostate	5	1.3		Z15.03
Genetic susceptibility to breast cancer	4	1.0	V84.01	Z15.01
Genetic carrier of other disease	3	0.8		Z14.8
PMS2-related Lynch syndrome (HNPCC4)	3	0.8	V84.09	Z15.09
BRCA gene mutation positive in male	3	0.8	V84.01, V84.09, V84.03	Z15.01, Z15.03, Z15.09
BRCA1 gene mutation positive in female	3	0.8	V84.01, V84.02, V84.09	Z15.01, Z15.02, Z15.09
BRCA2 gene mutation positive in male	3	0.8	V84.01, V84.03, V84.09	Z15.01, Z15.03, Z15.09
Other genetic carrier status (V83.89)	2	0.5	V83.89	Z14.8
Genetic predisposition to breast cancer	2	0.5	V84.01	Z15.01
Abnormal genetic test	2	0.5	795.2	R89.8
Carrier of gene for Lynch syndrome	2	0.5	V83.89	Z14.8
BRCA1 gene mutation positive in male	2	0.5	V84.01, V84.03, V84.09	Z15.01, Z15.09, Z15.03
Genetic predisposition to malignant neoplasm of breast	1	0.3	V84.01	Z15.01
Genetic susceptibility to ovarian cancer	1	0.3	V84.02	Z15.02
Genetic predisposition to ovarian cancer	1	0.3	V84.02	Z15.02
Genetic predisposition to disease	1	0.3	V84.89	Z15.89
BRCA1 genetic carrier	1	0.3	V84.01	Z15.01, Z15.09
Genetic susceptibility to other disease	1	0.3		Z15.89
Breast cancer, BRCA2 positive, unspecified laterality (HCC)	1	0.3	174.9, V84.01	C50.919, Z15.02, Z15.09
Other genetic carrier status	1	0.3	V83.89	Z14.8
Monoallelic mutation of PMS2 gene	1	0.3	V84.09	Z15.09
PMS2 deficiency	1	0.3	758.5	Q99.8
MSH6-related endometrial cancer (HCC)	1	0.3	182	C54.1
MSH6-related Lynch syndrome (HNPCC5)	1	0.3	V84.09	Z15.09
BRCA gene mutation test positive	1	0.3	V84.01	Z15.01, Z15.09
Familial hypercholesterolemia due to heterozygous low density lipoprotein (LDL) receptor mutation	1	0.3	272	E78.01
Familial hypercholesterolemia due to homozygous low density lipoprotein (LDL) receptor mutation	1	0.3	272	E78.01
Summary				
specific diagnoses (grey highlighted)	93	24.2	NA	NA
non-specific diagnoses	292	75.8	NA	NA

### Changes in Care due to Notification

Visually examining patient timelines for the 85 female HBOC patients (Figure 2A), we found 10 patients (12%) who appeared to have a change in care. Seventy-five female HBOC patients (88%) exhibited no change in care, of which 40 either had prior cancer or prior knowledge of their HBOC status. Among the 49 LS patients (Figure 2B), four (8%) did not have prior colon cancer and appeared to have received a colonoscopy related to their notification. Forty-five LS patients (92%) exhibited no change in care, of which 10 had prior cancer or prior knowledge of their LS status. Among the 66 FH patients (Figure 2C), LDL levels improved (at least temporarily) for six (9%) of the patients. Sixty (91%) of the patients positive for FH variants exhibited no change in care, of which 10 had CVD prior to both the notification of their FH status and the age of 50 years.

**FIGURE 2:**
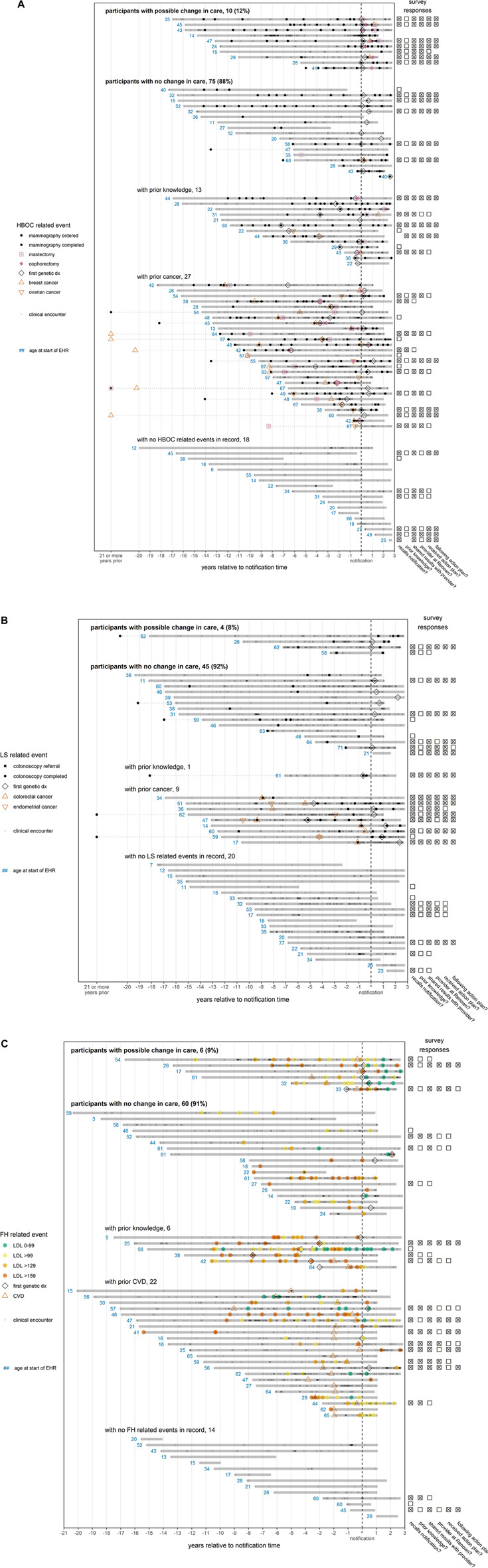
**(A) (B) (C)**—EHR timelines and survey responses for participants notified of findings. Each solid horizontal line represents a distinct participant medical record, with the duration of the medical record relative to the participant’s notification date indicated by the span of the line. A patient’s medical record is defined to begin with patient’s first record (procedure, diagnosis, or clinical encounter) and to end at the maximum date of the database (8/23/2021) or 1.5 years after the patient’s last record, whichever comes first. If an event recorded in the notes occurs outside of this timespan, it is linked to the remainder of the patient record with a dotted line. To preserve space, any event in the notes occurring at least 21 years prior to notification is marked on the *x*-axis as occurring “21 or more years prior”. The “first genetic dx” is the first time that a diagnosis indicating a variant associated with a given condition appears in a patient record. Points indicating CVD (ischemic heart events, cerebrovascular events, or peripheral vascular disease) or cancer (breast, ovarian, colorectal, or endometrial) are plotted at the earliest date a diagnosis was recorded. Since some diagnoses indicate a history of CVD or cancer, the disease may have been present earlier in the patient timeline. The red numbers indicate the age in years of a patient at the first event related to a patient’s finding, which is defined as a genetic diagnosis (all conditions); mammography, breast or ovarian cancer, or mastectomy (HBOC, panel **(A)**; colonoscopy, or colorectal or endometrial cancer (LS, panel **(B)**; CVD diagnosis or LDL test (FH, panel **(C)**. For FH (panel C), LDL test colors indicate the concentration of LDL in mg/dL. If available, survey responses are displayed to the right of each patient’s timeline. Questions answered affirmatively (“Yes”) or ambivalently (“Not sure” or “I don’t know”) are marked with an “x”, while survey questions answered negatively (“No”) are marked with an empty box. Questions not answered are left blank. From the left column to the right column, the questions are as follows: (1) “Did you receive positive genetic findings from the Healthy Nevada Project?”, (2) “Were you aware of your genetic variant prior to participating in the Healthy Nevada Project?”, (3) “Have you shared your results with any of your healthcare providers?”, (4) “Are any of the providers you shared your results with a Renown/Hometown Health associated provider?”, (5) “Did your provider design an action plan for you to follow?”, (6) “Are you currently following the action plan suggested by your provider?”. Patient records are grouped according to apparent participant responses to notification in their EHR. For HBOC (panel A), records are considered to exhibit a possible change in care after notification if there was an increase in the frequency of mammographies, or if there was a mastectomy/oophorectomy not preceded by cancer. For LS (panel B), records are considered to exhibit a possible change in care if there was an increase in the frequency of colonoscopies. For FH (panel C), records are considered to exhibit a change in care if LDL levels decreased (at least temporarily) to target levels (<100 mg/dl) after notification. For all conditions, participants with no change in care who had both prior presentation of disease (cancer or CVD) and prior knowledge were grouped according to whichever came first.

**FIGURE 2:**
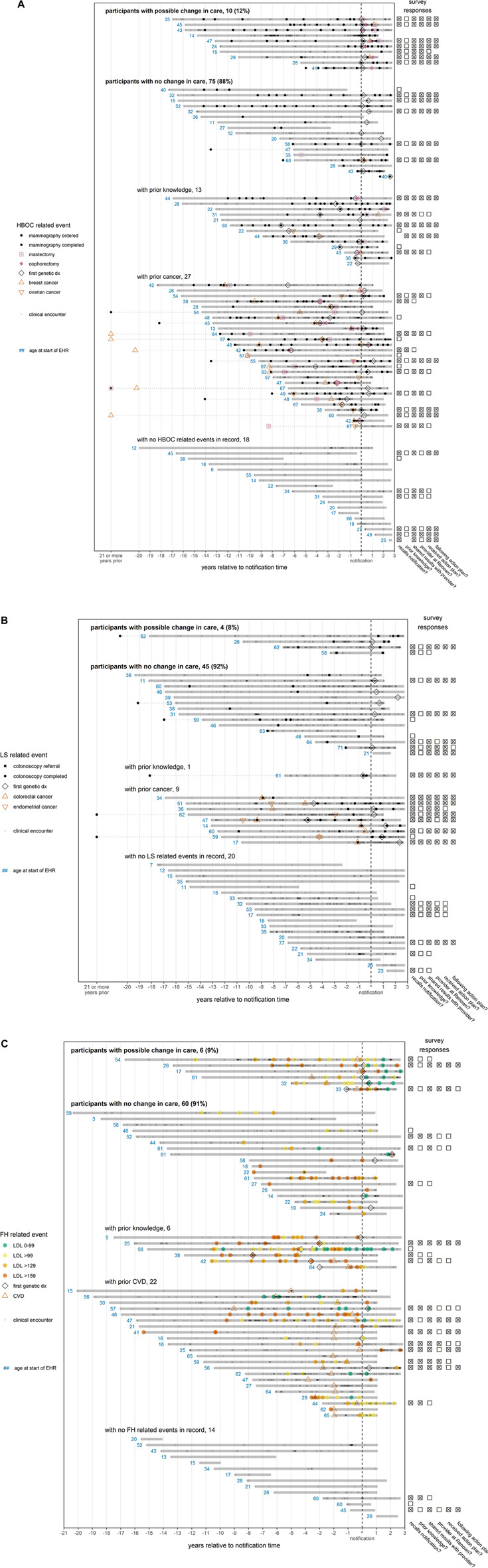
Continued.

**FIGURE 3:**
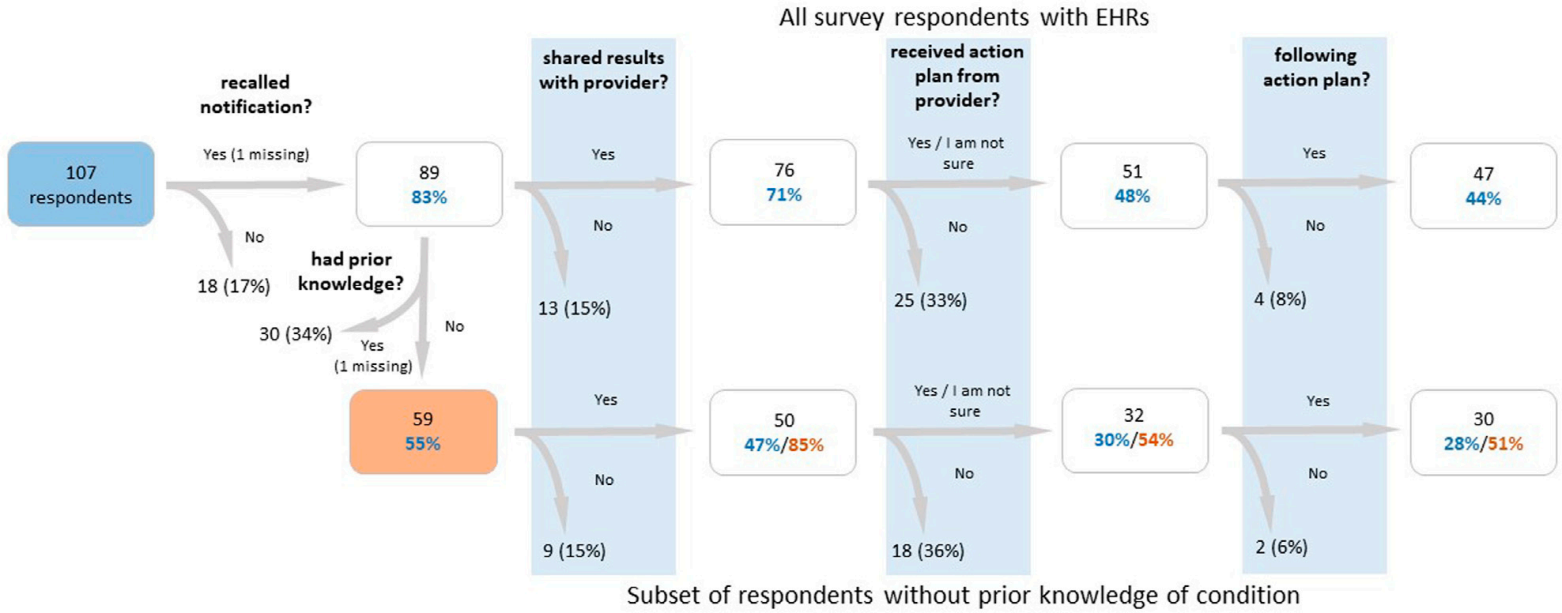
Flow chart of participant survey responses for participants with a finding, who were notified of their finding, and who had an electronic health record. The lower branch of the flow chart examines participants who did not have prior knowledge of their findings, while the upper branch examines all participants. Percentages in blue text are calculated out of the total number of respondents, percentages in red text are calculated out of the number of respondents who reported no prior knowledge (or whose response was missing for that question), and percentages in black are calculated out of the number of respondents in the previous box. From the left to right, the survey questions are as follows: (1) “Did you receive positive genetic findings from the Healthy Nevada Project?”, (2) “Were you aware of your genetic variant prior to participating in the Healthy Nevada Project?”, (3) “Have you shared your results with any of your healthcare providers?”, (4) “Did your provider design an action plan for you to follow?”, (5) “Are you currently following the action plan suggested by your provider?”.

The survey sent to T1Pos-notified (Supplementary Material S2) had an overall response rate of 39.6% and a 42.8% response rate among individuals with an EHR, for a total of 107 respondents with an EHR (Figure 3). Among these 107 respondents, 18 (17%) indicated that they did not recall being notified, and 76 (71%) indicated that they reported their findings to their healthcare providers. Of the 76 who reported their findings, 42 indicated that an action plan was formulated for them with 40 of those indicating that they were following their plan. Nine indicated they were not sure whether a plan was formulated for them; however, seven of those indicated they were following their plan. Altogether, 62% indicated that they were following their plan. 26 (34%) indicated that they had prior knowledge of their T1pos status (but only 11 of these had prior EHR documentation), and at least 45 (59%) indicated that they reported their findings to a Renown-affiliated provider. Of these 45, 18 (40%) had a diagnosis in the PL. Of the 59 participants that indicated no prior knowledge of their T1pos status, three (5%) had EHR documentation of their finding that preceded notification and 50 (85%) indicated that they reported their findings; none of the nine participants who did not report their findings (15%) had documentation in their EHRs (*p* = 0.02, Fisher exact, 2-tail). 22 (44%) of the 50 without prior knowledge and who reported their findings had a relevant nDx in their EHR, but only 13 (26%) had a relevant nDx in their PL. 81 (91%) of 89 respondents who recalled being notified indicated that they had shared or planned to share their T1pos results with their family members. Differences in responses to the survey between the three T1pos conditions were not statistically significant.

### Additional Results

The review of the clinical notes indicated that one participant notified their provider of their finding but specifically requested for it not to be documented. Their finding subsequently does not appear in their EHR.

According to data from HNP’s third-party vendor for 94 of the participants, only 18 participants (19%) sought confirmatory testing while 48 (51.1%) declined confirmatory testing during the initial counseling session. One confirmatory test resulted in no finding. However, among all notified participants with EHRs, 49 (19.6%) had a referral for a LGC and 32 (15.1% of those without prior knowledge) had a referral after notification.

Since our study period coincided with the COVID-19 pandemic, we also reviewed the frequency of encounters and procedures for the 250 notified participants with EHR to ensure that our findings were not affected by a persistent decline in healthcare services. Supplementary Figure S1 shows that other than a temporary decline in procedures and encounters at the beginning of the pandemic, healthcare utilization levels for participants in this study rebounded after several months to pre-pandemic levels.

## Discussion

The initial HNP model of returning CDC Tier 1 results was to empower the participants with their results. This “hands-off” approach relied on participants to act after notification and counseling. This approach provides an opportunity to examine how T1pos participants respond to notification and how efficiently those responses are recorded in the EHR and acted upon. Thus, the results of this analysis could provide a guide to other projects that are returning genetic results, and thereby enhance the effectiveness of population-based genetic screening (PbGS) in general.

Many studies that examine the outcomes of delivering actionable genetic findings to previously undiagnosed individuals do so in a clinical setting, and the clinical documentation of the finding is a given (Godino et al., 2016; Menko et al., 2019; David et al., 2021). It cannot be assumed that participants will act upon the information entrusted to them, even when the information is potentially life altering (as clearly indicated in their initial informed consent) and the individual is provided with professional advice regarding recommended action. This is especially true when testing was unsolicited as part of a research project, but even when testing was solicited, responses to pathogenic genetic findings may be influenced by an individual’s culture, family interactions, and life philosophy (Press et al., 2000; Binetti et al., 2006; Godino et al., 2016; Bijlsma et al., 2018). For instance, an individual’s balance between desire for control versus belief in fate may play a significant role in their response (Zimmermann et al., 2020). Additional factors such as age or prior presentation of T1pos related disease (such as breast cancer for an individual with HBOC) play a role as well. For these and other reasons, it has been shown that the uptake of pre-symptomatic genetic testing is considerably lower than 100% even for at-risk individuals (Finlay et al., 2008; George et al., 2015; Menko et al., 2019; Actkins et al., 2021; David et al., 2021). Considering that 9.1% of surveyed T1pos participants with an EHR record had prior, EHR-documented knowledge of their condition and additional participants already had interventions due to prior presentation of their underlying risk, it is perhaps not surprising that just 71% of T1pos Participants with EHR record indicated that they shared their results with their healthcare providers (Figure 3).

### Shortcomings of Documentation in the EHR

Although most participants shared their pathogenic genetic screening T1pos results with their healthcare providers, we observed a much lower rate of documentation of those results in their EHR (Figure 1). Survey results indicate that participants’ sharing their previously unknown genetic finding with their provider increases the likelihood of its documentation in the medical record. However, even when a participant says they have shared their results, less than 11% of such participants had a sufficiently specific diagnosis in their PLs. Since the PL is the primary method for indicating and sharing a patient’s active health problems between providers, these low documentation rates in the PL are especially worrisome. The discordance between sharing the results with providers and recording the finding in the EHR was not due to participants’ reluctance to have the finding documented and thus argues that the “hands-off” approach is not necessarily problematic but would benefit from overcoming some of the gaps in knowledge providers have with genetic testing and clinical decision support of genetic testing positive findings. The low EHR documentation rate does not appear to be due to participants’ reluctance to have the finding documented. It occurs despite significant promotional efforts within Renown in support of the HNP.

Even when findings were recorded, quite often diagnoses were not as specific as they could have been, considering the available nDxs in the EHR system. A diagnosis of “Genetic susceptibility to other malignant neoplasm” (Z15.09, 54 instances, Table 2) is too vague to inform clinical action. Similarly, recording “Genetic susceptibility to malignant neoplasm of ovary” (Z15.01) as a single code to document a finding of *BRCA1* or *BRCA2* does not convey the scope of the risk (as *BRCA1* and *BRCA2* also increase the risk of cancer of the breasts and other organs). Such non-specific coding may prevent appropriate risk-reduction interventions from being implemented. However, codes documenting specific variants were occasionally used (Table 2), indicating that more specific nDxs are available to providers.

We also observe cases where specific codes were used for documenting FH without the support of required clinical criteria. “Familial hypercholesterolemia” diagnosis (ICD-10-CM E78.01) is mostly used for patients without documented genetic findings of FH or evidence that a clinical criteria such as the DLCN was applied, thus reducing its significance, and necessitating the recording of a genetic variant for a provider to be certain that a patient was FH-T1pos. However, we could only find two such records for T1pos participants with FH.

The frequent use of non-specific diagnoses may simply reflect the widespread use of ICD-10-CM codes for clinical documentation and their relative inappropriateness for documenting genetic findings (Topaz et al., 2013; DeAlmeida et al., 2014; Fung et al., 2014). In contrast to ICD-10-CM, SNOMED CT^6^ has specific codes for *BRCA1* or *BRCA2* variants (SNOMED CD IDs 412734009/412738007 respectively). The use of non-specific diagnoses may also reflect documented issues in current EHRs with effective integration of genetic data with patient medical records (Kho et al., 2013) as well as issues with template designs, such as having to select codes from exhaustive lists.

However, another possibility may be that healthcare personnel are uncomfortable dealing with genetic testing and the resulting findings. Numerous studies have shown that healthcare personnel, especially in the primary care setting, do not feel adequately equipped to order genetic tests or interpret, communicate, and follow up on such results (Overby et al., 2014; George et al., 2015; Hamilton et al., 2017; Hann et al., 2017; Briggs et al., 2018; Hauser et al., 2018; Laforest et al., 2019; Menko et al., 2019; Demeshko et al., 2020). Reservations regarding insurance discrimination and the social impact the findings might have for the patient play a role as well, although we note that only one person in the results herein asked to have no mention of the finding in the medical record. Additionally, physicians may not pay attention to unsolicited genetic results within EHRs (eMERGE (Gottesman et al., 2013; Williams et al., 2019; Nestor et al., 2021)) and it may be unclear to healthcare personnel who is responsible for positive genetic testing results (Pet et al., 2019). Ours was not a usability study and we cannot attribute the relative weight of the factors that may contribute to the observed poor documentation. Nevertheless, it is likely that if integrated clinical decision support tools were available for the PCPs seeing patients with CDCT1 findings, better documentation rates would follow. Such tools might suggest the appropriate diagnostic codes for the condition, the risk and the genetic variant detected, as well as recommended follow up steps and intervals.

### Importance of Testing Early

Although we could not demonstrate improved practice patterns following T1Pos-finding notification for most participants (Figures 2A–C), many of the participants failed to benefit due to their old age, prior knowledge of their condition, prior presentation of outcomes, and prior interventions related to their findings. It is also possible that, because of the voluntary nature of the HNP, participants tend to be more health conscious than the general population and that this paradoxically contributed to our inability to detect improved practice patterns. Nevertheless, our results suggests that the timing of the genetic testing was a key factor. Had genetic screening been conducted earlier in life, many more participants would have benefited from T1Pos notification. Other studies (including our previous HNP publication) have reported similar findings (Grzymski et al., 2020; Guzauskas et al., 2020; Patel et al., 2020).

Since genetic testing after the presentation of a disease is clearly suboptimal, mandated testing in younger adult populations should be considered as a possible solution. In Nevada, a recently signed bill (SB251 (Nevada Legislature, 2021)), based on 42 U.S.C. 300gg-13 (GOVINFO, 2010), requires PCPs to obtain genetic counseling in compliance with the USPSTF recommendation (US Preventive Services Task Force, Owens et al., 2019) for risk assessment and possible genetic counseling and testing for all women with “a personal or family history of breast, ovarian, tubal, or peritoneal cancer or who have an ancestry associated with breast cancer susceptibility”. Even though the USPSTF recommendations were published in 2019, our review of the EHR indicates that widespread genetic screening under those circumstances is not yet common practice, especially if there was no evidence of relevant family history. Others have reported similarly low rates (Cham et al., 2022). Mandates such as Nevada’s may help identify many individuals at a younger age, prevent additional malignancies, and expand the scope of prevention by cascade testing. However, without sustained educational efforts within the general and medical communities, these types of efforts are more likely to increase screening after the presentation of symptoms rather than improve the ascertainment of family history in the medical record that will yield much earlier detection and risk reduction.

### Similar Studies

We are not aware of directly comparable studies attempting to measure the clinical outcomes of a “hands-off” return of results approach. The most similar study is probably Buchanan et al. (2020), which reported on the clinical outcomes of Geisinger’s genomic medicine experience (Williams et al., 2018), and their clinical data extraction and evaluation methods were similar. They provide similar information concerning diagnostic documentation and risk management, but in a different clinical setting and initiative design. In their report, post-disclosure diagnoses were evident in the EHRs of 13.4% of participants without prior knowledge, a rate comparable to the rate we observed in the PL. However, Buchanan *et al.* reported a much higher rate of post-disclosure risk management activities (70.2%) than we observed in our study. This may be because the definition of risk management activities used by Buchanan *et al.* for the T1pos conditions was significantly more encompassing (especially for FH) than our definition of behavior change. Nevertheless, the most likely cause of the difference in outcomes between our study and Buchanan *et al.* is the more integrative and proactive design of the Geisinger initiative.

### Limitations

A limitation of our study was the structure of healthcare in northern Nevada, where some subspecialties are predominantly private practice groups that have not provided us with access to their medical records. However, our review of the clinical notes indicated that procedures outside the reach of Renown’s Epic EHR are often documented in clinical notes during subsequent visits at Renown. Thus, even if a participant’s PCP was not an affiliated Renown physician and user of Renown’s Epic EHR system, it is reasonable that a significant genetic finding would eventually appear in the EHR record, given the typical rate of encounters at Renown and follow up time. We believe that the partial availability of clinical data due to the gradual implementation of Renown’s EHR from 2006 to 2011 had a minimal effect with regards to the recorded date of the finding but no effect on its actual documentation. When possible, additional specific dates were incorporated upon review of clinical notes.

Our review of EHR data was conducted at least 10 months after T1pos notification by the HNP. This was deemed sufficient time to allow T1pos participants to share their results with their physicians and for the findings to appear in the medical record. The existence of private practice groups was also the reason that for procedures such as colonoscopies, we considered orders as well as completed procedures. Although Renown’s coverage of primary care is roughly 50% in northern Nevada, at least 59% of survey respondents who shared their results shared with a Renown provider, suggesting a higher capture rate in our population. Although this was a single center study, the training and practice of medicine are comparable to other integrated networks and medical centers and our results should be considered in that broader context.

### Additional Observations

While our survey was not designed to evaluate how likely participants were to share their results with different types of family members, more than 90% of respondents indicated that they shared their finding with family. This level of uptake is comparable to the highest levels reported by others (Menko et al., 2019).

From the limited data set obtained from the third-party vendor that provides the genetic counseling, it is worth noting that more than half declined confirmatory testing and only 19% completed confirmatory testing. Thus, it seems that there is little value in recommending confirmatory testing. Financial or insurance considerations did not appear to be a significant contributing factor to the low rate of confirmatory testing. It may have been that HNP assurances regarding the robustness of the genetic testing results negated the importance of seeking confirmation for some participants.

The COVID-19 pandemic overlapped with our study period. We examined the possibility that this might have reduced participant utilization of healthcare, and thus affected our ability to detect responses to notification in the EHR. However, after a 2–4-month period of decreased utilization at the beginning of the pandemic, utilization rebounded to pre-pandemic levels (Supplementary Figure S1). Given that the minimum observation time was at least 10 months, we believe the pandemic had a minimal effect on our ability to detect responses to T1pos notification.

Only 60% of T1pos consenting participants with EHR were successfully notified and counselled, but the HNP has observed that the notification success rate was significantly higher when participants were contacted by Renown physicians than when they were contacted by the third-party vendor. This is likely due to Renown’s name recognition by participants. However, the third-party vendor success rate appears to be comparable to the rest of the industry. This highlights the need to find much more effective ways of reaching out to T1pos participants. Lack of notification was also associated with being non-white, who are underrepresented in the HNP (Table 1). This is likely a reflection on the socioeconomic disparities of certain non-white ethnic groups in northern Nevada^7^, negatively affecting their communication means and access to healthcare. Modifications to the HNP protocol including integration of the study into the EHR and improvements to the clinical decision support available to Renown Health providers will help address these disparity gaps moving forward.

### Conclusion

As a result of these findings and in conjunction with the new state law, SB251, Renown and the HNP have made significant changes including obtaining informed consent to report positive findings directly into the medical record of the consented patient. We have expanded physician and other provider education, created order sets within the EHR specific to the CDC Tier 1 conditions, as well as study- and CDC Tier 1-specific tip sheets for providers.

Altogether, our findings indicate significant missed opportunity to maximize the benefit of the HNP voluntary population-based genetic screening and suggests that a change of design is required when it comes to the integration of the results into the participants’ medical record. Relying on participants to share their T1pos status with their healthcare providers appears to be inefficient, suggesting that a much more proactive approach should be taken. To improve results, we propose that participants’ consent be obtained at the time of recruitment for the study to automatically integrate T1pos findings with their EHR and to directly contact the participants’ healthcare providers. Persistent training of medical staff regarding CDC Tier 1 conditions is also needed to maintain a high level of awareness of the significance of such results and ensure appropriate documentation. Medical staff should use the most specific available codes and should document the findings in the PL. Failing to document findings in the PL could result in a loss of knowledge regarding the patients’ at-risk status for years to come. However, as Nestor et al. (2021) showed, even documented findings can often be ignored. This highlights the need for continued outreach to T1pos participants and especially their healthcare providers on follow-up steps and documentation that needs to be taken to effectively manage disease risk and to ensure optimal outcomes of PbGS.

## Data Availability

The original contributions presented in the study are included in the article/Supplementary Material, further inquiries can be directed to the corresponding author.

## Footnotes

1 Renown Health, Reno, NV, United States https://www.renown.org/about/.

2 Epic, Verona, WI, United States https://www.epic.com/.

3 International Classification of Diseases, Ninth Revision, Clinical Modification (ICD-9-CM).https://www.cdc.gov/nchs/icd/icd9cm.htm.

4 International Classification of Diseases, 10th Revision, Clinical Modification (ICD-10-CM).https://www.cdc.gov/nchs/icd/icd10cm.htm.

5 Genome Medical, South San Francisco, CA, United States https://www.genomemedical.com/.

6 SNOMED International. https://www.snomed.org/.

7 United states Census Bureau. https://data.census.gov/cedsci/. Washoe County, NV Tables: Personal Income—B19301A-G,I, Household Tenure—B25003A-G, I, Geographic Mobility—S0701, Educational Attainment—S1501, Uninsured—S2702, Internet by Household Income—S2801.
