# Supplementary material for: Incomplete Penetrance of Population-Based Genetic Screening Results in Electronic Health Record
Source: Front Genet. 2022 Apr 27;13:866169. doi: 10.3389/fgene.2022.866169 (PMC9091193; doi:10.3389/fgene.2022.866169)
Supplement: Supplementary file 1 [file DataSheet1.docx]

Supplementary Material

# Supplementary Material 1 – List of Native Diagnoses (nDx)

# Supplementary Material 2 – Survey

General Information

**Dear Participant, the Healthy Nevada Project is about improving your health and the health of your**

**family. We would like to learn more about the impact of the project and our process on our**

**participants. We would greatly appreciate your cooperation in responding to the following brief**

**questionnaire.**

1. Do you have HEALTH INSURANCE or did you have HEALTH INSURANCE at the time you were notified about positive results?

- Yes
- No
- I would rather not answer

2. Do you have a PRIMARY CARE PROVIDER (medical doctor) or did you have a PRIMARY CARE PROVIDER at the time you were notified about positive results?

- Yes
- No

3. Did you receive positive genetic findings from the Healthy Nevada Project?

- Yes
- No
- Contact & Shared Results

4. Which condition were you contacted about?

- Familial Hypercholesterolemia
- Hereditary Breast and Ovarian Syndrome
- Lynch Syndrome

5. Were you aware of your genetic variant prior to participating in the Healthy Nevada Project?

- Yes
- No

6. Who initially contacted you about your positive genetic health findings?

- Genome Medical COORDINATOR
- Renown Medical Doctor
- I am not sure

7. Have you had a genetic consultation after you were notified about your findings?

- Yes, with a Genome Medical Genetic Counselor
- Yes, with a Renown Physician
- Yes, I don't know who with
- No

8. Have you received an information package for your primary care provider and your family members?

- Yes
- No
- I am not sure

9. Have you learned about the significance of taking a proactive approach to your health because of your genetic health findings?

- Yes
- No
- I am not sure

10. Have you shared your results with any of your healthcare providers?

- Yes
- No
- Results Shared With & Action Plan

11. Which health care providers have you shared your results with? Please indicate all.

- My primary care provider
- My specialist (e.g. cardiologist, gastroenterologist, etc.)
- Other medical professional

12. Are any of the providers you shared your results with a Renown/Hometown Health associated provider?

- Yes
- No
- I don't know

13. Did your provider design an action plan for you to follow?

- Yes
- No
- I am not sure

14. Are you currently following the action plan suggested by your provider?

- Yes
- No

15. Do you intend to follow the plan?

- Yes
- No
- I am not sure

16. When do you intend to start following the plan?

17. What is preventing you from following the plan right now?

18. What is your reason to not follow the plan?

Other (please specify)

19. What is preventing you from sharing your results with your primary care provider?

- I don't have time
- I can't afford it
- I am not interested in working on my health
- It is too difficult for me
- I am worried about the repercussions
- I plan to share my results at a later date
- Other (please specify)

20. Have you shared any of this information with your blood-related relatives?

- Yes
- No

21. Which blood-related relatives did you share this information with?

- sister
- brother
- daughter
- son
- granddaughter
- grandson
- mother
- father
- aunt
- uncle
- grandmother
- grandfather
- cousin

22. What is preventing you from sharing this information with your blood-related relatives?

23. Would you like us to contact you about your positive findings again?

- Yes
- No

24. Is there anything else you would like to share with us about this process of receiving your Healthy Nevada Project results?

## Supplementary Figures


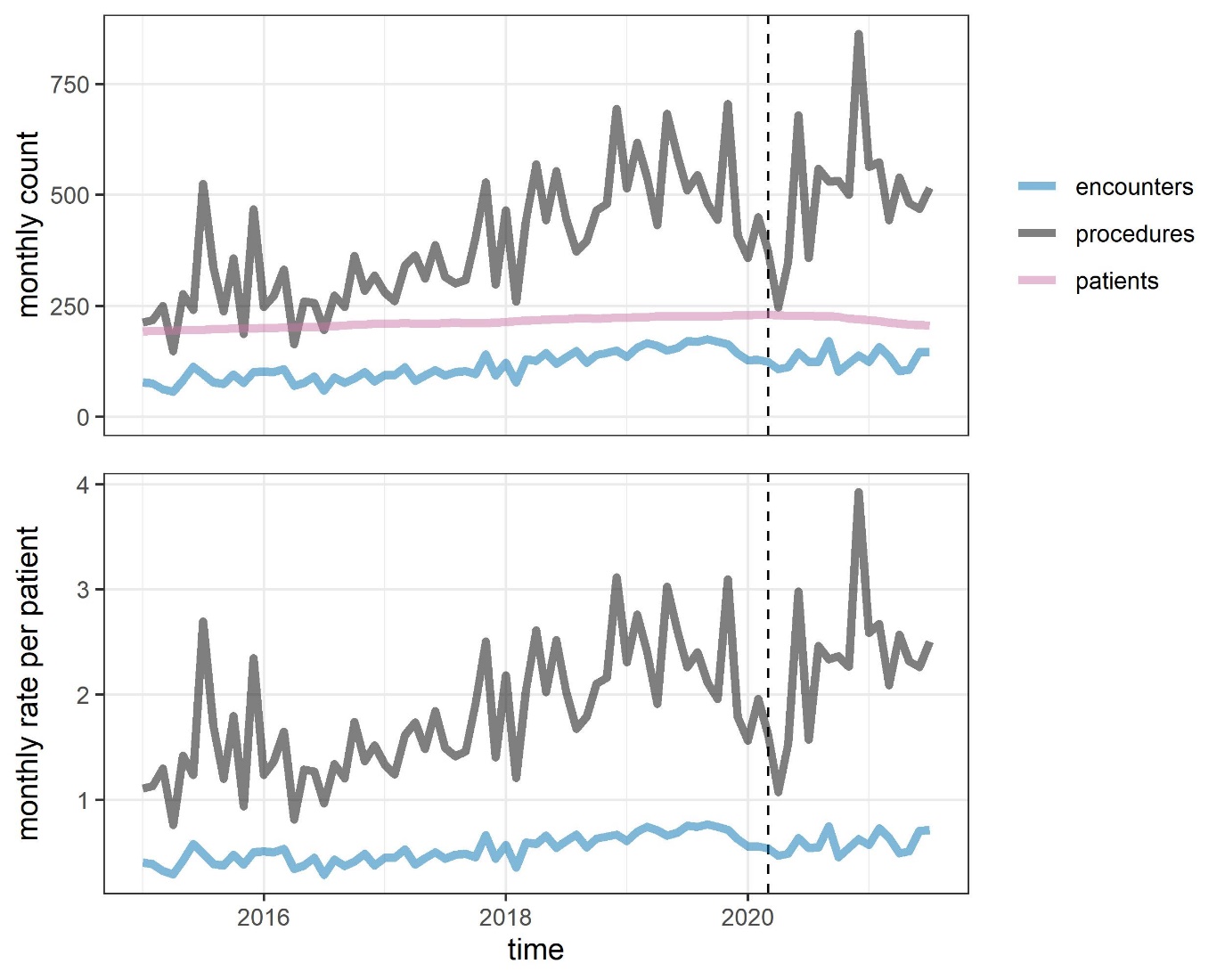


**Supplementary Figure 1 –** Healthcare utilization before and during the COVID-19 pandemic – Monthly counts (top panel) and rates (bottom panel) of encounters and procedures for participants with findings who were notified and had an EHR record. The purple line indicates the number of participants who had an active medical record in any given month. A participant’s medical record is defined to begin with a participant’s first record (procedure, diagnosis, or clinical encounter) and to end at the maximum date of the database (8/23/2021) or 1.5 years after the participant’s last record, whichever comes first. The vertical dashed line indicates March 2020, the start of the COVID-19 pandemic in the U.S.
